# Antiretroviral pause for HIV remission trials: A mixed methods study of people living with HIV in Soweto, South Africa

**DOI:** 10.1371/journal.pgph.0004425

**Published:** 2025-11-06

**Authors:** Fatima Laher, Mbalenhle Sibiya, Naledi Mahlangu, Kennedy Otwombe

**Affiliations:** 1 Perinatal HIV Research Unit, Faculty of Health Science, University of the Witwatersrand, Johannesburg, South Africa; 2 School of Public Health, Faculty of Health Sciences, University of the Witwatersrand, Johannesburg, South Africa; WUSM: Washington University in St Louis School of Medicine, UNITED STATES OF AMERICA

## Abstract

Trials may pause antiretroviral treatment to study HIV remission, but little is known about the perspectives of people living with HIV in highly prevalent regions. This mixed methods study qualitatively explored perspectives and quantitatively measured research participation willingness. Using snowball sampling, we included consenting adults with HIV in Soweto, South Africa. Of 100 people who completed questionnaires of demographics and participation willingness, 49 without analytic treatment interruption experience participated in 1 of 5 stratified focus groups, and 1 with analytic treatment interruption experience participated in an individual interview. We conducted the study at a research facility during 2024. The analyst performed manual inductive coding on transcripts from audio-recordings and analyzed thematically. We summarized quantitative data using descriptive statistics, compared groups using the Kruskal-Wallis test and assessed factors associated with willingness using univariate and multivariate logistic regression. Of 100 participants (44% female, median age 39 years) surveyed, 96% were willing to participate in HIV cure research, varying by study procedure: 75% if antiretrovirals paused, 97% if blood collected, 57% if lymph nodes, 41% if gut, and 39% if cerebrospinal fluid sampled. Willingness to pause antiretrovirals was similar for people aged 40+ versus 18–39 years (p = 0.248), antiretroviral duration 1–8 versus 9 + years (p = 0.759) and females versus males (p = 0.740). Prior cure research participation increased willingness to pause antiretrovirals (RR: 1.36, 95% CI: 1.17-1.58). The focus groups (n = 49) and interview (n = 1) revealed themes about pausing HIV treatment: physical and emotional health considerations, social considerations including non-adherence stigma and selective conditional disclosure, lifestyle optimization and healthcare interactions. Recommendations for researchers conducting analytic treatment interruption trials in South Africa include: counselling about monitoring, lifestyle health and treatment re-initiation; offering disclosure support; communicating directly with antiretroviral clinics about treatment pauses; and minimising mandatory invasive procedures. Negative healthcare experiences suggest treatment programmes should address staff burnout.

## Introduction

HIV remission, or “functional cure”, aims to achieve sustained viral suppression without the need for continuous antiretroviral therapy (ARV), to reduce side effects and burden of lifelong medication, and to improve health outcomes of people living with HIV. Although there are currently no proven remission strategies, it is a promising research field investigating approaches such as early ARV initiation, therapeutic vaccines, monoclonal antibodies, and chemotherapeutics [1].

The field faces a key scientific challenge of reducing latent HIV reservoirs, which early treatment can help achieve [2], and a development challenge of finding scalable, safe, and lasting interventions. Although stem cell transplants have induced durable remission in a few individuals, they are not deemed scalable or safe [3]. Moreover, the field faces equity challenges, with most HIV remission trials having been conducted primarily in the United States and Europe even though most people living with HIV reside in Africa [4]. Yet HIV remission research has had notable success in Africa. This includes a South African child from the CHER trial who initiated early ARVs, then, after stopping treatment, maintained long-term viral control with no detectable replication-competent virus and immune measures similar to children living without HIV [5]. Furthermore, about a fifth of African adult women from the SPARTAC sub-study who initiated early ARVs went on to suppress viral loads for a median of three and a half years while pausing treatment [6].

The trials themselves are also challenging, often requiring antiretroviral treatment pause, frequent monitoring and sometimes invasive procedures [4]. Without another biological marker, remission trials may deliberately pause ARVs temporarily under monitored conditions to study whether the remission strategy can increase time to viral rebound, reduce viral setpoint, reduce the viral reservoir, and induce protective HIV-specific immune responses.

There are known biopsychosocial and ethical considerations to pausing ARVs – the only treatment proven to extend health and lifespan and reduce transmission. The potential risks necessitate careful consideration and robust support systems. Participants have described psychological distress [7]. People living with HIV have anticipated socio-economic challenges associated with the burden of frequent monitoring visits [8]. There are also transmission risks during viral rebound [9]. In one small study, a short pause did not change HIV-DNA levels in cells or blood, but sometimes genetically diversified HIV in the reservoir [10]. However, many studies have shown that analytic treatment interruptions can be conducted safely with negligible risk of adverse events and drug resistance [11], and that most participants achieve viral re-suppression after restarting treatment following analytic treatment interruption [12]. Unlike structured treatment interruption with fixed time durations off ARVs during which resistance developed without benefits [13], in analytic treatment interruption the decision to re-start ARVs is informed by intensive monitoring.

Between 2000 and 2017, 159 studies incorporated analytic treatment interruption [14]. HIV remission research is emerging in Africa, especially around investigating monoclonal antibodies. The HVTN 805/HPTN 093 study was conducted in Botswana, Malawi, South Africa, and Zimbabwe with 13 participants. It conducted an analytical treatment interruption with participants who had initiated early ARVs after having received the monoclonal antibody VRC01 or placebo. A minority of participants experienced mid-term remission, and there were no reported serious adverse events, no adverse events of grade 2 or more, nor HIV transmissions [15]. The HVTN 806/A5416/HPTN 108 phase 1 randomized trial is being conducted in Botswana, Malawi, and South Africa. Analytic treatment interruption follows administration of a combination of two monoclonal antibodies, 3BNC117-LS-J and 10-1074-LS-J, or placebo amongst ART-treated adults living with HIV [16].

Although persons living with HIV routinely undergo phlebotomy for treatment monitoring in care, some HIV cure studies may sample tissues to analyze the nature, location and viral latency mechanisms of reservoirs [17]. The reservoir remains a barrier to cure, and the mechanisms of HIV persistence during antiretroviral treatment are not yet fully understood. Invasive sampling may cause discomfort and potential harm. In South Africa, 29% of vaccine trial participants without HIV did not consent to sampling of anogenital mucosal secretions [18]. Regional data from Africa are scarce on participation of people living with HIV in studies involving lymph node, cerebrospinal fluid, and gut biopsies – which represent some of the tissue reservoir sites [19].

Understanding how people living with HIV perceive and contextualize remission research involving treatment pauses may inform inclusive participant-centered approaches that prioritize the well-being and autonomy of participants living with HIV [20]. We measured willingness to participate in HIV remission research because it constitutes a trial recruitment issue relevant to powering trials with sufficient sample size. Previous cross-sectional surveys have measured that up to 68% of people living with HIV were willing to participate in analytic treatment interruption [21,22]. However, those online surveys may have underrepresented lower resource settings where HIV prevalence is highest and internet connectivity is limited. Our mixed methods study, in Soweto, South Africa, aimed to explore the experiences and perspectives, and measure willingness about HIV remission research amongst diverse adults living with HIV in a resource-limited high-prevalence setting where HIV cure trials are emerging.

## Methods

### Study design

**Fig 1:**
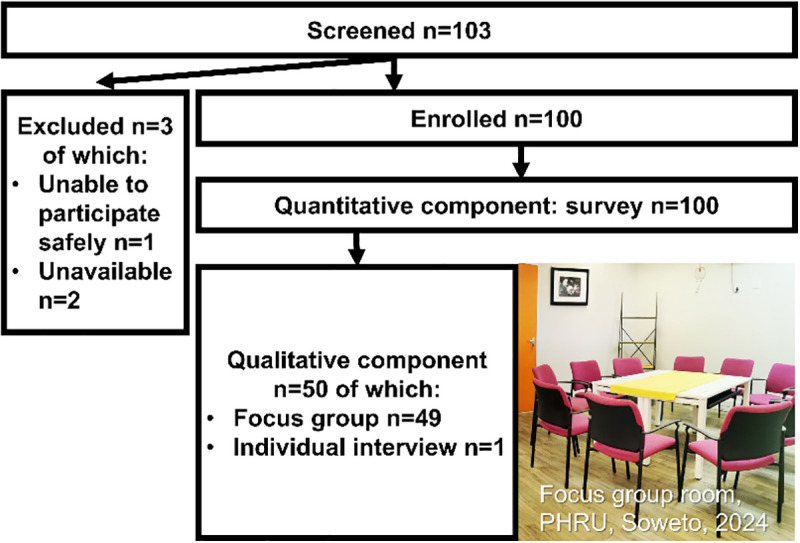
Flowchart of screening and enrolment into the data collection components.

We used a convergent parallel type of mixed-methods design: collection of quantitative and qualitative data was simultaneous, and analysis was separate (S1 GRAMMS Checklist). The results were combined during interpretation to inform each other. In the quantitative component, 100 people completed a once-off survey. A subset of 50 of them also participated in a once-off qualitative component of either a focus group or individual interview (Fig 1). Participants involved in both components completed the qualitative before the quantitative component, because the researchers intended to prevent exposure to concepts presented in the survey from shaping participant discussions.

Quantitative methods alone can measure willingness and show statistically significant differences between subgroups, but have limited ability to identify motivations, perceptions, and concerns that influence a major decision of pausing ARVs. Qualitative methods alone lack the generalizability and statistical power to quantify the prevalence of willingness within the sample and identify whether specific factors were associated. Thus, to avoid the limitations of a single method, we used a mixed methods design. We designed the quantitative component to provide measurable and more generalizable data about factors associated with willingness to undertake an analytical treatment interruption, and we intended that the qualitative component would elicit insight into experiences, perceptions, and beliefs that shaped analytical treatment interruption willingness. Although both components were focused on analytic treatment interruption willingness, the qualitative component solicited it broadly while the quantitative component focused on clinical or sociodemographic factors. Because the two sets of data were complementary, the qualitative and quantitative datasets were analyzed independently.

### Setting

Recruitment and study procedures occurred between May 2024 - August 2024 at the Perinatal HIV Research Unit in Soweto, a township in the Gauteng province where 72% of people are 15–64 years old, estimated HIV prevalence is 17.6% among 15–49 year olds [23], 88% live in formal dwellings and 16% attain higher education [24]. Gauteng is the most densely populated province in South Africa, where 81% of the population is Black African and 51% is female [24].

### Sampling strategies

Most participants were recruited by chain referral sampling because of HIV stigma. The exception was that for the individual interview, we applied purposive sampling by inviting an individual known to have achieved ARV-free remission, because they would have rich information about the experience; the researchers had prior information of one such case from a clinical trial.

### Eligibility

Inclusion criteria for all 100 participants consisted of individuals who self-reported living with HIV, were aged 18 years or older, and provided written voluntary informed consent. A subset of the first 49 participants who had never experienced HIV cure (eradication or remission) were invited to focus groups, and an individual interview was conducted with one participant who had experienced remission. Individuals were excluded from group or individual participation if they had any condition that challenged safe, meaningful participation.

### Qualitative research procedures

We planned five focus groups, based on methodological studies demonstrating the sufficiency of five focus groups to reach saturation for inductive thematic analysis [25]. We limited focus groups to 10 participants per group to optimize manageability, discussion depth and group dynamics while allowing contribution of diverse perspectives. We stratified the focus groups by gender and age to facilitate openness by minimizing power dynamics. Although methodological studies predict that 20 interviews are sufficient to reach saturation for individual interviews [26], there was only one person known to the researchers to have the experience of remission, which pragmatically limited the potential sample size.

#### Facilitation of focus groups and individual interview.

Only the two facilitators were present with participants during each focus group. The individual interview was conducted by one facilitator. During focus group discussions, one facilitator actively asked questions while the second ensured that, within the allocated time, all questions were asked and relevant responses were volunteered for each question. There was a pool of four multilingual facilitators who facilitated in English and commonly spoken local languages (isiZulu, Sesotho, Xhosa and Afrikaans) as required by participants. All facilitators were young female researchers with previous experience in HIV remission clinical trials and trained in qualitative methodology. Facilitators had no pre-study relationship with focus group participants, but they were on the trial team attending to the individual interview participant. Researchers told participants that the reason for doing the research is to help researchers understand how people think about acceptable ways to cure HIV. Guides for focus groups and individual interviews were similar, but individual interview questions were tailored for a person who had experienced remission, unlike focus group participants who had not.

#### Researcher reflexivity.

To mitigate potential biases, the researchers engaged in reflexive dialogs about their own biases about the research by discussing their interpretations with each other, exploring different perspectives and challenging assumptions. The researchers’ background conducting HIV (including remission) clinical trials may have shaped question framing and data interpretation toward the biopsychosocial framework.

#### Data collection.

Two trained multilingual facilitators conducted each focus group over a duration of up to 77 minutes and the individual interview over 15 minutes. Because of the technical nature of HIV cure concepts, we included the same brief information session in everyday language to orientate focus group and individual interview participants to the topic. After the participant consented and was determined eligible, the researchers showed a video of one of the researchers narrating the information (the script shown verbatim in Table 1 information session). Thereafter the researchers invited participants to ask clarifying questions and responded with explanations while refraining from providing additional information. The researchers chose video narration to help standardize the information given to all participants. The researchers chose a broad content structure to facilitate participant understanding of the key terms while minimizing biasing responses. The content of the information session was broad definitions of terms relevant to study questions: cure, eradication, remission and treatment interruption.

**Table 1:** Semi-structured guides for focus group discussions and individual interviews.

**Information session**	*In this session, when we say “cure”, we mean ending HIV in a person. Cure is not available right now, but is being researched in two main ways:* *i. HIV remission: researchers would try something to keep a person’s HIV controlled for a while so that they don’t need ARVs during that time. Remission means HIV is still in the body, but it is controlled (suppressed, undetectable in blood) without ARVs. We don’t know how long remission can last, so a person may need to test often to see if HIV comes back, and may need more remission treatments every time HIV comes back.* *ii. HIV eradication: this means that researchers would try something to get all HIV out of a person. This is more difficult because when we are infected, HIV mixes its own genes into our genes (DNA) in our cells.* *In research studies about curing HIV, a person who is taking ARVs would take an experimental treatment and afterward pause their ARVs for a while to see if the virus is still making copies. Usually, if a person stops ARVs, HIV makes copies and makes the body sick, and the virus copies can transmit to others.*	
**Post-information questions about HIV remission research attitudes**	**Focus groups**	**Individual interview**
	What do you think about HIV remission?	What do you think about the time you were in HIV remission?
	To understand if a cure idea is working, some researchers may ask people living with HIV to stop ARVs for a while. Tell us what you and other people you know who are living with HIV would think about while deciding if you would want to join a cure research study which asks people to stop ARVs for a while?	To understand if a cure idea is working, some researchers may ask people living with HIV to stop ARVs for a while. What did you think about when you were deciding if you wanted to join the remission cure research study which asked you to stop ARVs for a while?
	How would people react if you told them you were pausing ARVs for a time while they were in a cure research study?	How did people react when you told them you were pausing ARVs for a time while you were in the study about remission?

Thereafter, the facilitators used a semi-structured guide that had been refined during pilot testing to ask non-leading, open-ended questions and probes (Table 1). At the end, facilitators reminded participants that HIV cures are not widely available yet, so ARVs are the best option currently.

### Quantitative research procedures

#### Survey.

All participants self-completed a questionnaire of close-ended questions. The questionnaire assessed willingness, including actual participation in an HIV cure research study; willingness to participate in a future HIV cure research study; willingness to pause ARVs if part of an HIV cure research study; and willingness to participate in a cure research study that collects blood, cerebrospinal fluid, lymph gland, gut/intestine tissue. The questionnaire assessed socio-demographics of age, gender identification, highest education, and work status. The questionnaire assessed clinical HIV data including the year of ARV initiation, reasons for skipping ARV treatment for longer than one week, and presence of any non-HIV comorbidity.

#### Sample size.

The sample for the quantitative component of the study was 100 people. The sample size calculation was based on *a priori* power analysis for an outcome not relevant to this report.

#### Variables.

The primary outcome was willingness to pause ARVs.

### Data analysis

#### Qualitative data analysis.

The audio-records were transcribed verbatim and translated into English. We conducted a manual thematic analysis [27]. A trained analyst read the transcripts, then assigned codes to text excerpts. The codes emerged organically from the data (Table 2). Through discussions and re-readings of the transcripts, the analyst refined the codes and themes with two researchers, who had also been facilitators, until there was consensus. Quotations were chosen to illustrate the themes, with speaker anonymity maintained by attributing them to their focus group or individual interview. We present results of the focus group – all individuals who had never experienced remission – separately from the individual interview with the case who had experienced remission.

**Table 2:** Coding tree of codes and themes emerging from focus groups and individual interview.

Themes	Codes
Physical and emotional health considerations of pausing treatment	Individual differences predict outcomes Treatment maintains health and appearance Desire to escape pills and their side effects Treatment preference Monitoring and interventions for continued health stability Experience of non-adherence Treatment resumption concerns Emotional effect of happiness Emotional effect of fear
Social considerations of pausing treatment	Support from loved ones Non-adherence stigma Selective conditional disclosure
Lifestyle and behavioral optimization	Lifestyle optimization also optimizes HIV management Lifestyle optimization not preferred Suboptimal lifestyle choices while taking antiretrovirals Belief in need to optimize lifestyle for remission
Healthcare interactions	Health monitoring Concerns about negative healthcare provider reactions Researcher communication with healthcare provider Training of healthcare providers

#### Statistical analysis of quantitative data.

We summarized the data (S1 Data) using descriptive statistics, including measures of central tendency and dispersion for continuous variables, and frequencies and percentages for categorical variables. We evaluated overall willingness to participate in cure research, and willingness to pause ARVs in research. Comparisons between categorical variables were conducted using the Fisher’s exact test. For continuous measures, medians (interquartile ranges [IQR]) were determined and compared by willingness to pause ARVs using the Kruskal-Wallis test. We used univariate and multivariate logistic regression models, with a log-binomial link function to allow for assessment by relative risks (RR) and their associated 95% CI, to determine factors associated with a higher probability of willingness to pause ARVs for HIV cure research. We considered variables with a p-value <0.1 at the univariate level for inclusion in the multivariate model. We conducted statistical analyses using SAS Enterprise Guide version 7.15 (SAS Institute Inc., Cary, NC, USA).

### Ethics statement

The University of the Witwatersrand Human Research Ethics Committee (Medical) approved the study. All participants gave written, voluntary informed consent. For confidentiality, data are presented without identifying information.

## Results

### Qualitative results from people who had not experienced analytic treatment interruption and remission

#### Demographics.

For the focus groups, we telephonically prescreened 77 people and scheduled 50 people for screening based on their availability (13 were unavailable) and possible eligibility (14 would not have met criteria). Of the 50 screened individuals, 49 were eligible to participate, and 1 participant was excluded because of concerns about safe and meaningful participation in focus groups. Forty-nine people without experience of analytic treatment interruption and remission participated in one of five focus groups: n = 9 females 18–40 years old, n = 10 females 41 years and older, n = 10 males 18–40 years old, n = 10 males 41 years and older, and n = 10 non-binary persons 18 years and older.

#### Theme 1: Physical and emotional health considerations of pausing treatment.

There were interconnected sub-themes of physical health and emotional wellbeing.

Participants believed that the effects of treatment pause on physical health, including the chance to achieve HIV remission, would be individually variable because people had different bodies, nutritionally determined immune resilience, CD4 counts, and viral load control.

“Our bloods are not the same, and I wonder how remission would treat me.” [Non-binary, 18 years or older, living with HIV]

“I think HIV remission can happen to those whose viral load is, maybe it’s, 500 copies, not more than, so that they will be able to control it. If it is more than 1000, they cannot control it; they cannot suppress the HIV in your body.” [Female, 18-40 years old, living with HIV]

Participants anticipated a range of effects that treatment interruption may have on emotions. Some feared a treatment pause. They believed it had the potential to cause physical illness and change appearance.

“Do I still look the same after 3 months [of pausing treatment] or…?” [Non-binary,18 years or older, living with HIV]

“Only one month, and we’d fall to the ground. Now your skin is dry and unattractive to other people. Stopping even for a while, is defaulting. If you use pills, just use the pills and be yourself.” [Male, 41 years or older, living with HIV]

There were also fears about resuming treatment after a pause and regimen changes, reflecting participants’ awareness of initial side effects, viral resistance, and treatment failure. Some had a negative attitude toward uncertain health outcomes from treatment pause.

“Remission is giving the HIV virus strength to come back. Okay, the immune system is like this: ‘Let’s change!’ Because it is a smart virus, it changes and changes. Yeah, so remission? Ai no.” [Male, 18-40 years old, living with HIV]

“So when I come back from remission and I fail [treatment], where am I?” [Non-binary, 18 years or older, living with HIV]

“When we keep stopping and restarting, we will have to deal with side effects.” [Individual interview, female, experienced remission]

However, many thought interrupting treatment would bring them happiness because it would liberate them from pills.

“I’d be happy to know that my body can function by itself without being boosted by something.” [Male, 18-40 years old, living with HIV]

Some stated a preference for continuing daily ARVs instead of experimenting with a treatment pause in a remission study.

“It [taking treatment] has become my hobby, because I know there’s a certain time when I take my pills. So it will be hard when this remission is being done… My pill is my baby, it’s my sibling, it’s my friend: at a certain time, I sit with it.” [Male, 41 years or older, living with HIV]

“We are dependent on our ARVs so that when you fall ill, you at least know that you have soldiers assisting you on the side.” [Male, 18-40 years old, living with HIV]

However, others shared their experiences of surviving short-term non-adherence without physical illness as proof that a treatment pause should not be dismissed as necessarily dangerous.

“I am not proud of that [weekend non-adherence] and I am not saying it’s okay to do that, but I am saying this [treatment pause research] could be a good test for them [researchers] to see how they can do it – it’s a research, right?” [Male, 41 years or older, living with HIV]

The desire to escape treatment – including pill fatigue and treatment side effects – was a facilitator to consider participating in pause research studies.

“It’s tiring to always have to take pills.” [Female, 41 years or older, living with HIV]

Participants believed that pause research should optimize participant safety by regularly monitoring physical health and viral load, and provide interventions for continued health stability.

“So immediately you see something, you’re going to take me back to my ARVs. And I don’t think my viral load will skyrocket.” [Female, 18-40 years old, living with HIV]

“Before you start this thing [remission trial], maybe there is a supplement they give you so it can help you push, maybe for a certain period of time…” [Male, 41 years or older, living with HIV]

#### Theme 2: Social considerations.

Participants believed that the support of loved ones during a treatment pause could help them adhere to study requirements and cope with challenges. However, they also feared negative reactions of non-adherence stigma because of perceived irresponsibility, and lack of understanding from family members. Some participants expressed a desire to shield loved ones from worrying about potential health complications of stopping treatment by not disclosing. Some recommended that research clinics offer the option of helping to disclose a treatment pause to family members.

Participants discussed strategies for selective conditional disclosure to gain support from loved ones. They anticipated that they would feel comfortable disclosing an ARV pause to close family members only if those family members were already aware of their HIV status. They preferred to disclose to friends and their community only after achieving remission. Additionally, they were open to communicating with sexual partners if they were likely to be supportive, if they were already involved in their healthcare, and if they might be concerned about the potential risks associated with stopping treatment.

“He [partner] won’t have a problem. The only thing is that he would also want to join what I have joined.” [Female, 41 years or older, living with HIV]

“I will tell my family because I know they would support me no matter what. Just like when I told them that I’m positive, they showed me support – they told me to take treatment. So even now, if I tell them, I don’t think they would hold back their support. They would be supportive and say, ‘Yes, try and see if that study… maybe there might be success in finding the cure.’ So I would tell my family. Not my friends or my partner.” [Female, 18-40 years old, living with HIV]

“Yoh, my mom will start swearing at me, saying, ‘Even on the day when you are skinny, they [researchers] must pick you up’… When things go bad, she will say, ‘Go to them!’ She won’t be supportive the way I want her to be.” [Female, 18-40 years old, living with HIV]

A young female participant who described her social group drinking pills together every day feared that a treatment pause might isolate her from her social group.

The participant in the individual interview described the experience of receiving negative reactions, including non-adherence stigma, from loved ones, which she interpreted as their fear that she would become sick.

#### Theme 3: Lifestyle and behavioral optimization.

There was a belief that lifestyle optimization – involving nutrition, exercise, avoiding parties, alcohol restriction, smoking cessation and condom use – could help HIV management but restricted enjoyment of life. Participants stated their experiences about making suboptimal lifestyle choices, especially using alcohol, while taking ARVs, and they believed that the beneficial effects of ARVs on health and transmission made the chore of lifestyle optimization less necessary. All except the group with non-binary individuals volunteered their belief that lifestyle optimization would become necessary again to achieve and sustain HIV remission if one was pausing ARVs.

“We party, we drink, and as [another participant] said, sometimes you forget the pills when you go somewhere and then you skip taking it. And sometimes you end up having sex without a condom. So now when you are no longer on medication, you understand that it can risk your life.” [Female, 18-40 years old, living with HIV]

“Because the lifestyle we have while on ARVs – I don’t wanna lie to you – we abuse it. We don’t use condoms because we’re drinking pills. We drink, we smoke, we do all these things because we are depending on these pills for protection. So when it comes to this research but the person is not on treatment: they need to control how they behave.” [Female, 41 years or older, living with HIV]

#### Theme 4: Healthcare interactions.

Generally, participants expressed a preference for continuous health monitoring to ensure safety, address illness promptly, and evaluate effectiveness during a potential remission trial. There was a belief that researchers ought to have a backup plan if remission was not achieved. Some participants expressed the need for their healthcare during treatment pause to help maintain their health stability, e.g., another treatment, intervention or nutritional support for immune benefit. One participant said that frequent blood draws for monitoring remission sounded unpleasant.

“So, when they say you must stop your ARV, then there must be something that they give you to continue taking so that your body health stays stable. Something like, that will make your viral load remain low. Not, like, just leaving your treatment and you are just not taking any treatment.” [Female, 18-40 years old, living with HIV]

There were expressions of concern about healthcare providers’ potential negative reactions to a research participant pausing ARVs. Participants stated their experiences of nurses who reprimanded them for non-adherence and stigmatised them as defaulters. There was a belief that a person who paused ARVs for remission research may face similar reactions, as well as stigma for joining a study pausing ARVs for financial gain, and provider-generated barriers to accessing care and ARV re-initiation. Some participants described their experience of avoiding reprimand from nurses for missing medication by switching clinics and pretending to be a new patient each time. Others said that they did not experience reprimand but rather being traced at home whenever they did not take ARVs.

“The kind of nurses that we have in the neighborhood clinics are rude… behaving as though they rule the world. Like, when you get there after having defaulted... when you get there and you need their help, firstly, they will start by shouting at you, ‘Who said you must do that? You don’t listen to the things that we tell you…’, this and that. At that time, she is going up and down in the clinic going to other nurses and when she gets there, she would make you sit there for a long time and then when she comes back and finds you still sitting there, they say ‘Is she still here? I’m not the one who puts her in the situation that she’s in right now! She will wait for me!” [Female, 18-40 years old, living with HIV]

“…they [clinic staff] will remind me that they came looking for me, and I told them that I had joined a study and stopped drinking pills, so there’s nothing they can help me with.” [Male, 41 years or older, living with HIV]

Participants believed that healthcare workers would need training on new research and treatment options, such as remission, to ensure they can support participants effectively. There was a belief that if researchers directly communicated with healthcare providers, preferably in person or with a letter, nurses’ anger about research participants pausing treatment would be assuaged and continuity of correct care could be ensured. There was also a belief that healthcare workers would likely try to convince the participant to re-initiate instead of pausing ARVs.

“Researchers should go to the clinics and teach nurses and doctors [about remission studies] so that they are also alert.” [Non-binary, 18 or older, living with HIV]

“When they start with this research and all that, you [researchers] have to inform the hospitals because they help us live.” [Female, 41 years or older, living with HIV]

### Qualitative results of one case of a participant who experienced analytic treatment interruption and remission

#### Demographics and clinical information.

For the individual interview, we screened and enrolled one person, a female aged 18–40 years old. Before her HIV diagnosis, she had preventively received the experimental monoclonal antibody VRC01. She had maintained undetectable or near-undetectable viral loads on an analytic treatment interruption until week 117 when she resumed ARVs [15].

#### Theme 1: Physical and emotional health considerations of pausing treatment.

She said that, although she had always adhered to treatment before, her decision to join a study pausing treatment was motivated by the desire for relief especially from physical side effects of ARVs.

“It is that I was tired of taking ARVs, and that in the morning when I woke up, I would feel sick from the side effects of these pills. Yeah, I would have diarrhea for two weeks and sometimes I would lose weight you see... and it would affect my appetite.”

Her pre-existing fear of becoming physically ill from stopping ARVs subsided after researchers informed her that treatment pause would be temporary. She found hope in the strength of her body when her CD4 count was maintained during the pause. She displayed a positive attitude toward her perception that she regained her appetite and weight during the pause.

“What gave me a boost was the fact that I was not taking them [treatment] and since I stopped taking them, I gained weight a bit. And I saw my body fighting, and all those symptoms I used to have on my face, I no longer had them.”

The participant recalled experiencing the same two emotional states as predicted by those participants not in remission – happiness and fear – when she was in remission.

“I was happy.”

Despite being in remission, the participant experienced a feeling of fear caused by uncertainty about the cause of incidental illnesses, for example whether it was due to stopping ARVs or unrelated factors.

“I would sometimes feel down, why, because sometimes eish, I would wake up sick and not know if maybe it’s because I have stopped taking ARVs.”

Her fear also originated from a belief that HIV could return suddenly during a pause, causing her to become sick and die.

“It makes you afraid that eish... that means you can die at any time, you see?”

#### Theme 2: Social considerations.

The participant described instances when she had to manage family and partner expectations to remain on ARVs. She described that her loved ones initially did not support her decision to pause treatment, because they were afraid for her health. This made her decision making process more difficult.

“He judged me and told me about how when people don’t take their treatment have symptoms and become sick, the way they are quick to become sick. So I viewed that as him being judgmental towards me.”

Loved ones even tried to override her autonomy by telling her what decisions to make about research participation, but she interpreted it as their worry about her health.

“She told me to tell them [researchers] that I need to stop participating in this study because I was risking my life.”

She described that as she remained virally suppressed off treatment, her own fears dissipated and then her loved ones supported her decision to continue the analytic treatment interruption.

And when time went on, I explained to them that that fear that I had – that I was scared that if I don’t drink my pills, I will get sick – is no longer there. And that my body and my antibodies are stronger than I thought they were. So they accepted and supported me.”

#### Theme 3: Lifestyle and behavioral optimization.

Researchers had worked with the participant on optimizing nutrition and sleep for overall wellness. The participant said that the analytic treatment interruption optimised her use of condoms.

“To tell the truth, sometimes I didn’t use a condom, but this made me to go back to using condoms.”

#### Theme 4: Healthcare Interactions.

Researchers had accompanied the participant to her ARV clinic to communicate about analytic treatment interruption and related medical matters. The participant had a positive attitude toward her experience of trial counselling. Despite her own fears and loved ones challenging her autonomy, she thought that the respect and care she received from her research team facilitated her decision to interrupt treatment temporarily.

“I continued because I liked the way I was treated on the study.”

### Quantitative results

#### Socio-demographics.

We surveyed 100 participants living with HIV, 44% identified as female, 60% completed high school as their highest level of education, and 76% were unemployed. Median age was 39 (IQR 31–45) years.

#### HIV.

Among the 100 participants living with HIV, 98% were taking antiretroviral treatment and 15% had at least one comorbidity additional to HIV. Median time since ARV initiation was 9 years (IQR 6–16), and the median year of ARV initiation was 2015. 41% of participants reported ever skipping ARVs for longer than a week. The three most frequent reasons for non-adherence arose from pill fatigue (n = 19); psychological distress (n = 15); and social reasons including relationship issues, stigma, and cultural reasons (n = 13) (Table 3). Thirteen participants reported multiple reasons for non-adherence.

**Table 3:** Socio-demographic and clinical characteristics of 100 people living with HIV in Soweto, South Africa by stated willingness to pause ARVs during HIV cure research.

Characteristic	Total (n = 100)	Willing to pause ARVs for research (n = 75)	Unwilling to pause ARVs for research (n = 25)	P-value
***Socio-demographics***				
Age in years, median (IQR)	39 (31-45)	40 (31-46)	35 (30-41)	0.120
**Gender identification, n (%)**				
Female	44 (44%)	36 (48%)	8 (32%)	0.355
Male	40 (40%)	28 (37%)	12 (48%)	
Non-binary	16 (16%)	11 (15%)	5 (20%)	
**Highest education completed, n (%)**				
Primary school	25 (25%)	20 (27%)	5 (20%)	0.349
High school	60 (60%)	46 (61%)	14 (56%)	
Tertiary	15 (15%)	9 (12%)	6 (24%)	
**Employment**				
Employed, including self employed	24 (24%)	18 (24%)	6 (24%)	0.999
Unemployed	76 (76%)	57 (76%)	19 (76%)	
***HIV***				
Current ARV treatment, n (%)	98(98%)	73(97%)	25(100%)	0.999
Years since ARV initiation, median (IQR)	9 (6-16)	10 (7-16)	7 (5-15)	0.1830
Ever skipped ARVs for longer than a week, n (%)	41(41%)	32 (43%)	9 (36%)	0.642
Any comorbidity, n (%)	15(15%)	10 (13%)	5 (20%)	0.518
**Reasons for ARV non-adherence**				
Pill fatigue	19 (19%)	16 (21%)	3 (12%)	0.388
Psychological distress	15 (15%)	10 (13%)	5 (20%)	0.518
Social reasons including relationship issues, stigma, and culture	13 (13%)	10 (13%)	3 (12%)	0.999
Financial constraints	7 (7)	7 (9%)	0 (0%)	–
Adverse effects	7 (7)	7 (9%)	0 (0%)	–
Healthcare provider instructions	2 (2)	1 (1%)	1 (4%)	0.439
Stock shortage	2 (2)	2 (3%)	0 (0%)	–
Analytic treatment interruption in a research study	1 (1)	1 (1%)	0 (0%)	–

#### Willingness to participate in HIV cure research.

Of the 100 participants, 93% had never participated in HIV cure research before, and 96% indicated willingness to participate in a future HIV cure research study (Table 4). Although 97% were willing to participate in an HIV cure research study collecting blood, 57% were willing if the study collected lymph node tissue, 41% if the study collected gut tissue and 39% if the study collected cerebrospinal fluid. People identifying as females were more willing than males to participate in cure research that would collect cerebrospinal fluid (54.5% vs 27.5%, p = 0.015).

**Table 4:** Willingness to participate in HIV cure research amongst 100 people living with HIV by gender identification and by age.

Variable	Total n = 100	Gender identification				Age		
		Females n = 44 (%)	Males n = 40 (%)	Non-binary n = 16 (%)	P-value	18-39 years old n = 52 (%)	40 years and older n = 48 (%)	P-value
Previous participation in HIV cure research study	7	4 (9)	3 (8)	0 (0)	0.677	2 (4)	5 (10)	0.256
Willingness to participate in future HIV cure research study, n (%)	96	44 (100)	36 (90)	16 (100)	**0.036** ^¶^	49 (94)	47 (98)	0.619
That pauses ARVs	75	36 (82)	28 (70)	11 (69)	0.355	36 (69)	39 (81)	0.248
That collects blood	97	44 (100)	38 (95)	15 (94)	0.204	50 (96)	47 (98)	1.000
That collects lymph node tissue	57	28 (64)	18 (45)	11 (69)	0.141	31 (60)	26 (54)	0.687
That collects gut tissue	41	23 (52)	13 (33)	5 (31)	0.140	22 (42)	19 (40)	0.840
That collects cerebrospinal fluid	39	24 (55)	11 (28)	4 (25)	0.022*^¶^	20 (39)	19 (40)	1.000

**NB:** Where global p-values were significant¶ , pairwise comparisons were conducted comparing females vs. males*, females vs. non-binary** and males vs. non-binary***.

#### Willingness to pause ARVs during HIV cure research.

75% indicated willingness to pause ARVs during an HIV cure research study (Table 4). There were no statistically significant differences by age and gender in previous participation and willingness to participate in HIV cure research. The proportion willing to pause ARVs during a cure study in people aged 40 years and older versus those aged between 18–39 years old was not significantly different [39/48 (81%) versus 36/52(69%), p = 0.2476). Similarly, it was not significantly different in those whose treatment duration was 1–8 years versus 9 or more years [31/44 (71%) versus 44/56 (79%), p = 0.759] and in people who identified as female versus male [36/44 (82%) versus 28/40 (70%), p = 0.740].

More females than males stated willingness [100% (n = 44) versus 90% (36/40), p = 0.032*] to participate in future HIV cure research. More females than males [55% (24/44) versus 28% (11/40), p = 0.012*] and the non-binary [55% (24/44) versus 25.0% (4/16), p = 0.040**] stated willingness to undergo cerebrospinal fluid collection as part of future HIV cure research (Table 4).

#### Factors associated with willingness to pause ARVs during HIV cure research.

In the multivariate regression analysis, the only variable associated with a higher likelihood of being willing to pause ARVs in a research study was previous participation in HIV cure research (RR: 1.36, 95% CI: 1.17-1.58) (Table 5).

**Table 5:** Factors associated with willingness to pause ARVs in a research study.

	Univariate		Multivariate	
Variable	RR (95% CI)	p-value	RR (95% CI)	p-value
**Age 40+ versus 18–39 years**	1.17 (0.94-1.47)	0.166		
**Gender identification**				
Male versus female	1.17 (0.91-1.50)	0.214		
Non-binary versus female	0.98 (0.67-1.45)	0.927		
**Highest education completed**				
Primary versus secondary school	1.04 (0.82-1.33)	0.729		
Tertiary versus secondary school	0.78 (0.51-1.21)	0.271		
**Employed versus unemployed**	1.00 (0.77-1.30)	1.000		
**ARV treatment duration ≥9 years versus 1–8 years**	1.12 (0.88-1.41)	0.364		
**Ever skipped ARVs for longer than a week versus never**	0.91 (0.73-1.14)	0.407		
**Any comorbidity versus none**	0.87 (0.60-1.27)	0.475		
**Previous participation in HIV cure research**	1.37 (1.20-1.55)	<.0001	**1.36 (1.17-1.58)**	**<0.0001**
**Willing (versus unwilling) to participate in HIV cure research study that**				
Collects lymph node tissue	1.07 (0.85-1.35)	0.566		
Collects gut tissue	1.26 (1.01-1.56)	0.037	1.19 (0.95-1.49)	0.134
Collects cerebrospinal fluid	1.2 (0.99-1.52)	0.061	1.13 (0.90-1.41)	0.284
**Non-adherence reason**				
Pill fatigue	1.16 (0.91-1.46)	0.228		

## Discussion

In summary, our study in Soweto, South Africa, showed that about three-quarters of adults living with HIV are willing to undertake analytic treatment interruption research, driven by the hope for liberation from daily medication but tempered by physical health fears, social implications, and practical considerations. Fewer participants were willing to undergo invasive study procedures. Only previous research participation was statistically associated with willingness.

Our results differ from a study in Ghana in which people living with HIV were reluctant about analytic treatment interruption, mainly because of illness fears [28]. Also different from our study, the reasons that some people living with HIV in Australia were willing to participate in HIV cure trials hinged upon social reasons [29,30]. Geographical context is thus relevant.

In our study, the common experience of non-adherence may partly explain the willingness to undertake analytic treatment interruption. Both the qualitative and quantitative components corroborated that the desire to escape pill burden and side effects motivated people to consider pausing ARVs. This echoed the actual experience of the participant who had achieved remission and found relief from ARV-related physical symptoms. Our quantitative data measured that two-fifths of our participants had self-paused their ARVs unmonitored for longer than a week, and that pill burden was the most common reason for ARV non-adherence. In the year 2022, 18.6% of the 5 730 647 people in South Africa taking antiretroviral therapy were virally non-suppressed above 1000 copies per ml [31]. Suboptimal adherence in South Africa has been associated with socio-economic, patient, health system, and treatment factors [32]. The implication is that the considerations about time off ARVs – implicit to analytic treatment interruptions – are already familiar to many people living with HIV. This illuminates the gap in durable solutions to optimize the quality of life of people living with HIV: there is a need for alternatives to daily pills, for example longer-acting ARV chemotherapeutics and remission treatments, in addition to improving psychosocial support.

Participants in our study raised similar considerations about analytic treatment interruptions to other populations. Despite willingness, participants feared physical deterioration, viral rebound and treatment failure. In Australia, people living with HIV were also concerned about health deterioration and viral rebound, but also transmission [29,30]. In the United States of America, young adults living with HIV were concerned about physical and psychological effects of antiretroviral interruption, the possibility of having their HIV status revealed to others because of participation, and the time required to participate. Their participation facilitators included regular laboratory monitoring and sexual partner protection [8]. In an older South African study, people preferred interrupting treatment only if cure was likely [33], but in our study some were willing to consider pausing treatment with uncertain outcomes.

The person in our study who had experienced remission raised similar themes to the 49 participants who had not experienced analytic treatment interruption nor remission, corroborating the psychological distress fearing physical illness, the non-adherence stigma and the need to optimize behaviors. This was different to a study in the United States of America where these were not main concerns, but the description of stress mitigation through researcher rapport is similar [34]. Our qualitative data demonstrated the relevance of emotional and social context factors to participants’ willingness to pause treatment for research. Participants in focus groups feared non-adherence stigma and potential judgment from loved ones and healthcare providers, with some considering selective disclosure to mitigate these risks. This apprehension is validated by the lived experience of the individual who achieved remission, whose family and partner initially expressed concern and attempted to talk her out of it because they feared for her health. The participant’s eventual family support, following her sustained viral suppression, underscores the importance of understanding from social networks.

To our knowledge, this is the first study that has documented a belief that changes in nutrition, alcohol, smoking and exercise would be preferable to mitigate risks of an analytic treatment interruption. Previous research has documented that people living with HIV perceive that sexual behaviour change would be necessary during treatment pause [35]. Our study participants did not raise explicit concerns about HIV transmission to partners during analytic treatment interruption. This is different to a study conducted in the USA in which key informants – though people living with HIV were not included – perceived the need for partner prevention measures, specifying pre-exposure prophylaxis, vaginal rings and condoms [36]. Our participants framed partner protection mostly as condom use. We understand this within the context of the current unavailability of vaginal rings in this setting, and by a study in South Africa which found that oral pre-exposure prophylaxis is not amongst preferred prevention methods because it is neither long acting nor discreet [37]. Our study findings suggest that people living with HIV may choose not to disclose their participation in analytic treatment interruption research to partners who are not supportive. This could reduce opportunities for partner referral and provision of prevention care.

Our findings that females have slightly higher willingness – overall and for an invasive procedure – suggests that female underrepresentation in HIV cure research [38] is not because of unwillingness to participate. Trial eligibility criteria around contraception, pregnancy and lactation may reduce female inclusion. Although these criteria safeguard against potential vertical transmission during analytic treatment interruption, sufficient representation of women remains crucial to understand how remission strategies affect female bodies, for example with regards to hormones [39].

### Recommendations

Our findings suggest recommendations for advancing HIV cure trials with treatment interruptions in South Africa (Table 6). Participants emphasized robust safety measures, participant information, social support and non-stigmatizing healthcare interactions as important practical elements for supporting participation in analytic treatment interruption.

**Table 6:** Actionable findings and recommendations for analytic treatment interruption researchers in South Africa.

Finding	Recommendations for researchers
1. People living with HIV are aware of treatment adherence requirements and benefits	Treat potential remission trial participants as capable and competent to exercise autonomy on decisions to participate, pause treatment and heath behaviors during pause.
2. Non-adherence stigma	Offer support to disclose analytic treatment interruption to families and partners Obtain permission and offer to accompany participants to notify ARV providers about trial participation, explain the research, offer copies of the signed informed consent forms for their records and request that antiretroviral management be transferred to researchers for the trial duration. This may be an opportunity to legitimize the pause, alleviate participants’ fear of treatment provider backlash, share pertinent medical history, prevent wasted dispensing during treatment pause, and build relationships to facilitate post-pause reintegration into treatment.
3. Desire to pause pills co-exists with fears	Inform that pause is temporary. Inform about intensive monitoring (CD4, viral load and health status) and re-starting ARVs before significant decline. Inform about measures to minimize viral resistance risks.
4. Desire for wellness	Inform about pause monitoring and treatment restart. Counsel about exercise, alcohol reduction, tobacco cessation, and condom use. Consider offering nutritional assistance if food insecure or not properly nourished.
5. Pill fatigue	Consider long-acting injectables, and if unavailable or inappropriate, the simplest possible oral regimen. Counsel about social support (e.g., people at home and adherence clubs), setting small rewards as a motivation. Discuss side effects and consider regimen change. Conduct mental health screening. Solicit their preferred adherence strategy, e.g., multi-month dispensing, alarms, pill box.
6. Invasive procedures reduce participation willingness	Study designs should avoid mandatory invasive procedures. Research must continue to seek alternatives to invasive procedures

After a brief orientation, people living with HIV in Soweto were able to weigh risks and benefits of ARV pause for remission research based on their pre-existing awareness that non-adherence to ARVs had consequences on viral load, CD4, physical health, longevity, transmission and treatment sensitivity. Our findings support existing practices of comprehensive informed consent processes that address risks, benefits, as well as emotional and practical considerations surrounding analytic treatment interruption [40]. Current monitoring requirements in trials with analytic treatment interruption are intensive [41], but our data show that people living with HIV favorably perceive the emphasis on safety. People living with HIV also perceive a need for lifestyle optimization during analytic treatment interruption, suggesting that counselling should address nutrition, exercise, stress management, and risk reduction behaviors.

Invasive procedures may be a barrier to participation for people living with HIV who are considering cure trials. Whenever possible, invasive procedures should be optional and not mandatory for participation in the main trial. Although initially 96% of people living with HIV in our study indicated willingness to participate in a future HIV cure research study, willingness dropped to between 39–57% when invasive sampling (cerebrospinal fluid, gut, lymph nodes) were specified. A qualitative study conducted in the United States of America found that many people living with HIV were opposed to invasive procedures, such as cerebrospinal taps and bone marrow biopsies, largely due to concerns about the potential pain and discomfort involved [42]. Researchers are exploring alternative methods to invasive sampling, including 18F-fluorodeoxyglucose Positron Emission Tomography (FDG-PET) imaging [43].

The theme of social support supports the recommendation that researchers should offer to help participants navigate disclosure challenges and build supportive networks. Selective disclosure is a common strategy for individuals navigating life with HIV. Our study supports the findings of earlier research in the region in which community members supported the autonomy of people living with HIV about whether to inform sex partners about participating in an analytic treatment interruption trial [44].

We also recommend that researchers communicate directly with healthcare providers. Obtaining permission from cure trial participants to share information with their ARV clinic might assist coordination and transparency. Given overwhelming evidence for treatment efficacy, healthcare providers, communities and even researchers may understandably feel bound by the paradigm of continuous ARVs and reluctance to pause ARVs. Regardless, the ethical obligation to ensure informed consent is different from a paternalistic restriction on people living with HIV to participate in cure research. In a South African study in Durban, 20 general community members had doubts that people living with HIV should interrupt treatment for cure research [44]. The latter finding should caution well-meaning stakeholders to avoid gatekeeping the autonomy of people living with HIV who are considering analytic treatment interruption study participation, and also caution medical professionals against the educational elitism of dominating decision-making. Our study demonstrates how African people living with HIV evaluate concerns about pausing treatment toward making informed decisions. Ethically, people living with HIV retain autonomy to participate in cure research benefiting their communities. Progress of cure research will need support from health-care providers, communities, and researchers, not non-adherence stigma.

Even outside of research settings, our recommendation to equip healthcare providers to support participants with non-stigmatizing care is relevant. Although we did not set out to investigate it, our study documents a negative consequence on people living with HIV who sacrifice continuity of care to avoid staff exhibiting distress about non-adherence. Compassion fatigue and microaggressions may signal arising occupational burnout in established ARV programs [45]. Although burnout is often viewed from a staff lens, for example a fifth of HIV healthcare workers felt emotionally exhausted [46], future research should also investigate and address causative systemic factors including workload, healing environments, and stakeholder relationships. Healthcare staff burnout could plausibly create barriers to participation of people living with HIV in remission research.

### Strengths and limitations

A strength of our study design is that we included the rare perspective of an African woman who experienced remission in an analytic treatment interruption trial. Few studies have documented actual experiences of analytic treatment interruption. In the United States of America, 14 participants, mostly males, described altruism as their motivator, and experienced both positive and fear-based emotions during analytical treatment interruption [7,47]. In our study, the participant’s motivation was to obtain relief from pills, and she also described feeling simultaneous happiness and fear, as anticipated by others in our study who had not paused treatment. Her experience of social repercussion paralleled anticipated social concerns of people who had not paused treatment analytically. These parallels again confirm the importance of biopsychosocial support during analytic treatment interruption. However, this same strength of our study design was also a limitation, necessitated by the single known existing case study in our geographic area. We thus have incomplete understanding of the experience of analytic treatment interruption and remission in South Africa.

Size and diversity are strengths of our study, with 100 participants across the gender and age spectrum, 50 of them in the qualitative component. In the Netherlands study, the views of 20 men in an acute HIV infection cohort were documented [48], and similarly some were willing to interrupt treatment, but had concerns about viral rebound transmission. And in the United States of America, 20 young adults living with HIV described motivators as altruism, stigma reduction, and relief from clinical burdens of HIV, clinical monitoring and partner protection, but barriers as potential physical side effects, psychological distress, disclosure, and participation time [8].

## Conclusions

In the South African context, HIV cure researchers should directly coordinate with ARV clinics, support pause disclosure, and communicate plans for health maintenance.

## Supporting information

S1 Data Excel file of dataset. (XLSX)

S1 GRAMMS Checklist Word file of GRAMMS checklist for mixed methods. (DOCX)
