# Supplementary material for: Antiretroviral pause for HIV remission trials: A mixed methods study of people living with HIV in Soweto, South Africa
Source: PLOS Glob Public Health. 2025 Nov 6;5(11):e0004425. doi: 10.1371/journal.pgph.0004425 (PMC12591421; doi:10.1371/journal.pgph.0004425)
Supplement: S1 GRAMMS Checklist — (DOCX) [file pgph.0004425.s002.docx]

Good Reporting of A Mixed Methods Study (GRAMMS) checklist

| **Guideline** | **Section-subsection** |
| --- | --- |
| Describe the justification for using a mixed methods approach to the research question | Methods-Study design |
| Describe the design in terms of the purpose, priority and sequence of methods | Methods-Study design |
| Describe each method in terms of sampling,  data collection and analysis | Methods-Sampling strategies  Methods-Qualitative research procedures  Methods-Quantitative research procedures  Methods-Qualitative data analysis  Methods-Statistical analysis of quantitative data |
| Describe where integration has occurred, how it has occurred and who has participated in it | Methods-Study design |
| Describe any limitation of one method associated with the present of the other method | Methods-Study design |
| Describe any insights gained from mixing or integrating methods | Discussion |

O'Cathain A, Murphy E, Nicholl J. The quality of mixed methods studies in health services research. J Health Serv Res Policy. 2008;13: 92-98.
